# The effects of intranasal esketamine (84 mg) and oral mirtazapine (30 mg) on on-road driving performance: a double-blind, placebo-controlled study

**DOI:** 10.1007/s00213-017-4706-6

**Published:** 2017-07-28

**Authors:** Aurora J. A. E. van de Loo, Adriana C. Bervoets, Loes Mooren, Noor H. Bouwmeester, Johan Garssen, Rob Zuiker, Guido van Amerongen, Joop van Gerven, Jaskaran Singh, Peter Van der Ark, Maggie Fedgchin, Randall Morrison, Ewa Wajs, Joris C. Verster

**Affiliations:** 1 Utrecht Institute for Pharmaceutical Sciences (UIPS), Division of Pharmacology, Utrecht University, Universiteitsweg 99, 3584CG, Utrecht, the Netherlands; 2 Institute for Risk Assessment Sciences (IRAS), Utrecht University, Utrecht, the Netherlands; 3 Nutricia Research, Utrecht, the Netherlands; 4 Centre for Human Drug Research (CHDR), Leiden, the Netherlands; 5 Janssen Research & Development, LLC, Raritan, NJ USA; 6 Janssen Research & Development, Janssen Pharmaceutica N.V, Beerse, Belgium; 7 Centre for Human Psychopharmacology, Swinburne University, Melbourne, Australia

**Keywords:** Driving, SDLP, Esketamine, Mirtazapine, Depression

## Abstract

**Rationale:**

The purpose of this study is to evaluate the single dose effect of intranasal esketamine (84 mg) compared to placebo on on-road driving performance. Mirtazapine (oral, 30 mg) was used as a positive control, as this antidepressant drug is known to negatively affect driving performance.

**Methods:**

Twenty-six healthy volunteers aged 21 to 60 years were enrolled in this study. In the evening, 8 h after treatment administration, participants conducted the standardized 100-km on-road driving test. Primary outcome measure was the standard deviation of lateral position (SDLP), i.e., the weaving of the car. Mean lateral position, mean speed, and standard deviation of speed were secondary outcome measures. For SDLP, non-inferiority analyses were conducted, using +2.4 cm (relative to placebo) as a predefined non-inferiority margin for clinical relevant impairment.

**Results:**

Twenty-four participants completed the study. No significant SDLP difference was found between esketamine and placebo (*p* = 0.7638), whereas the SDLP after mirtazapine was significantly higher when compared to placebo (*p* = 0.0001). The upper limit of the two-sided 95% confidence interval (CI) of the mean difference between esketamine and placebo was +0.86 cm, i.e., <+2.4 cm, thus demonstrating that esketamine was non-inferior to placebo. Non-inferiority could not be concluded for mirtazapine (+3.15 cm SDLP relative to placebo). No significant differences in mean speed, standard deviation of speed, and mean lateral position were observed between the active treatments and placebo.

**Conclusions:**

No significant difference in driving performance was observed 8 h after administering intranasal esketamine (84 mg) or placebo. In contrast, oral mirtazapine (30 mg) significantly impaired on road driving performance.

## Introduction

Despite efforts to improve automobile safety and decrease unsafe driving practices, road trauma remains a serious public health problem (World Health Organization 2009). Psychoactive drugs that affect the central nervous system have the potential to impair driving performance (Walsh et al. 2008). Various factors leading to road accidents after taking psychoactive drugs involve poor vehicle control, impairment of basic driving skills, and impaired decision-making, which increase the risk of accidents during driving (Corazza et al. 2012; Mozayani 2002; Schifano et al. 2015).

The anesthetic and sedative use of ketamine is well-documented. Intranasal ketamine is an anesthetic of choice for patients with acute injury in moderate-to-severe pain in emergency conditions (Shrestha et al. 2016; Yeaman et al. 2014). Also, rapid antidepressant effects can be achieved using intranasal ketamine with low treatment-associated adverse events (Lapidus et al. 2014). Anesthetic as well as sub-anesthetic concentrations of ketamine have shown to produce neuropsychological effects and rapid mood-enhancing actions in electroconvulsive therapy for treatment-resistant depression, while anesthetic concentrations result in greater magnitudes of antidepression and cognitive protection (Zhong et al. 2016). The psychotomimetic effects of ketamine are dose-dependent and are avoidable when used at maximal anesthetic doses (Järventausta et al. 2015). The effect of ketamine on depression will also be reached at doses lower than the typical anesthetic doses, and at that low dose level, side-effects are generally mild and transient (Bobo et al. 2016).

Ketamine’s psychomimetic effects including modulation of cognitive processes, emotional responses, memory, and learning (Aalto et al. 2005; Duan et al. 2013; Seeman et al. 2005) could be attributable to its activity on cholinergic systems (postanesthetic delirium) (Hustveit et al. 1995), memory learning attention (Kohrs and Durieux 1998), or adrenergic and dopaminergic systems (Vollenweider et al. 2000; White and Ryan 1996). However, the exact mechanism of action of ketamine contributing to these effects is not well-known and thus remains to be elucidated. Ketamine is a racemic mixture consisting of two enantiomers, R(−) and S(+) ketamine (Zeilhofer et al. 1992). Esketamine (s-[+]-ketamine enantiomer) is a non-competitive, subtype non-selective, activity-dependent *N*-methyl-d-aspartate (NMDA) receptor antagonist which has threefold to fourfold higher affinity for NMDA receptors and threefold anesthetic potency compared with the R-(−)-ketamine enantiomer (Kohrs and Durieux 1998; Oye et al. 1992; Vollenweider et al. 1997). The antidepressant effect of esketamine is thought to result from preferential blocking of NMDA receptors on rapid firing inhibitory GABA-ergic interneurons, which in turn enhances the activity of glutamatergic neurons by increasing the presynaptic release of glutamate and stimulation of postsynaptic α-amino-3-hydroxy-5-methyl-4-isoxazolepropionic acid (AMPA) receptors (Duman et al. 2012; Sanacora et al. 2008). Together, these actions potentiate the release of BDNF and activation of its downstream neurotrophic intracellular signaling pathways that result in increased synaptic protein synthesis and synaptogenesis, ultimately restoring synaptic function (Duman et al. 2012; Sanacora et al. 2008). Furthermore, the psychomimetic effects of esketamine have been attributed to its potential to release dopamine in the striatum (Hashimoto 2017).

The anesthetic state of patients with detachment from the environment and self under ketamine effect was best described by the term “dissociation” (Järventausta et al. 2015). The non-medical use of low-dose (0.15 mg/lb–0.33 mg/kg) ketamine either as an inhalation or i.v. injection can cause mild dissociative effects, visual and auditory hallucinations, and feeling of distortions of time, space, and reality. Moreover, high doses (2.2 mg/kg) may induce severe dissociation, known as a “K-hole,” in which individuals experience detachment from reality and severe distortions in consciousness (Muetzelfeldt et al. 2008), neurobehavioral performance deficits (sustained and divided attention) (Passie et al. 2005), reduced reaction time, and subjective assessments of alertness (Micallef et al. 2002) for up to 3 days after ketamine use. Of note, subjective feelings of permuted perception could prove lethal as far as driving is concerned (Giorgetti et al. 2015; Muetzelfeldt et al. 2008).

In Hong Kong, available reports have documented 45% ketamine positivity in intoxicated drivers involved in non-fatal traffic accident (Wong et al. 2010) and 9% positivity in fatal crashes (Cheng et al. 2005). Neurocognitive and psychomotor deficits associated with ketamine use reduce an individuals’ ability to effectively and simultaneously interpret and organize incoming visual, auditory, and tactile information and concurrently impede appropriate behavioral reactions, which may result in car crashes due to increased lane deviation, reduced reaction and braking time, and greater steering deviations (Hayley et al. 2015). Due to sparse observational roadside drug studies, controlled examination of sub-anesthetic doses of ketamine or other drugs of abuse on driving performance is required for further validation (Stough et al. 2012). The 2012 US package insert for ketamine states that patients should be cautioned that driving an automobile, operating hazardous machinery, or engaging in hazardous activities should not be undertaken for 24 h or more (depending upon the dosage of ketamine hydrochloride and consideration of other drugs employed) after anesthesia. It is not clear to which extent this 24-h period is supported by data or chosen arbitrarily. Moreover, as esketamine is administered at a sub-anesthetic dose, the period during which the subject would not be able to drive a car or operate a machine could be shorter compared to after its use as an anesthetic.

The objective of this study was to evaluate the effect of intranasal esketamine compared to placebo and a positive control mirtazapine, on driving performance using the mean difference of the standard deviation of lateral position (SDLP) parameter of an on-the-road driving test.

## Methods

### Study design

This was a single-center, double-blind, double-dummy, randomized, three-way crossover study in healthy men and women. The study comprised an eligibility screening examination (between 21 days and 1 day prior to the first dose administration), a three-way cross-over double-blind, a single dose treatment phase, and a follow-up examination (within 7 to 10 days after the last dose administration). Between each test day, a washout period of at least 6 days was scheduled. The study was approved by the BEBO Medical Ethics Committee, and subjects were paid for participation in the study. The study was sponsored by Janssen Research and Development, and conducted in collaboration between the Centre for Human Drug Research, Leiden, the Netherlands (clinical assessments), and Utrecht University (driving tests).

### Participants

Twenty-six healthy men and women, aged 21 to 60 years, who met all inclusion and none of the exclusion criteria, were enrolled in this study. Subjects had to have a valid driving license for more than 3 years, have driven at least 5000 km in the past year, and were to be driving a car on a regular basis. They were included if they had a body mass index (BMI) between 18 and 30 kg/m^2^ and a body weight no less than 45 kg. Subjects had to have normal visual acuity (corrected or uncorrected). Subjects with mental or physical disease were excluded, and the use of psychoactive medication known to affect driving performance was not allowed. Subjects were tested for drugs of abuse and alcohol use at entry in the clinical research center. Subjects who withdrew from the study were replaced in order to have the requisite 24 participants who completed the study.

### Treatments

To ensure blinding, treatments were administered using a double-dummy technique: on each test day, participant received both an intranasal and oral treatment. On each test day, subjects received one of the following treatments: (1) intranasal esketamine (84 mg) and oral placebo, (2) intranasal placebo and oral mirtazapine (30 mg), or (3) intranasal placebo and oral placebo. Intranasal esketamine was supplied in a nasal spray pump. The device delivered 16.14 mg esketamine hydrochloride (14 mg esketamine base) per 100-μL spray. Intranasal placebo was supplied by the sponsor in a nasal spray pump. The placebo device delivered 0.1 μg of denatonium benzoate per 100 μL spray. During screening, participants were trained to use the intranasal device. Oral mirtazapine was supplied as over encapsulated tablets of 30 mg mirtazapine. Oral placebo was supplied as color- and size-matched capsules.

### Study procedures

In the morning, subjects reported at the Center for Human Drug Research in Leiden.

Before administration of the treatment, participants were not allowed to eat for 2 h, and not to drink any fluids for 30 min. Standard safety assessments included physical examination, vital signs, 12-lead ECG, clinical chemistry, hematology, urine pregnancy tests (for women of childbearing potential only), and urinalysis. Study medications were self-administrated (under medical supervision) in the morning at approximately 11:30 or 13:00 h. Subjects remained in the clinic, and received a light meal approximately 2 h after dosing. The on-road driving test was scheduled in the evening, 8 h after treatment administration. The potential effects of intranasal esketamine on dissociative symptoms, psychosis, and suicidal ideation and behavior were evaluated by the use of rating scales (Clinician Administered Dissociative States Scale [CADSS], 4-item positive symptom subscale of the Brief Psychiatric Rating Scale [BPRS+], and the Columbia Suicide Severity rating Scale [C-SSRS]) that were completed by the investigator. Approximately 1 h before the scheduled start of the driving test, participants were transported from the clinic to Utrecht. Just before transportation, i.e., 6 to 7 h after treatment, a blood sample was taken to determine plasma concentrations of esketamine, its metabolite noresketamine, and mirtazapine. Driving tests were conducted at 7.30 p.m. or 9 p.m., approximately 8 h after treatment administration. After completion of the driving test, subjects were transported back to the clinic in Leiden, where adverse events were reported.

### The on-road driving test

**Fig. 1:**
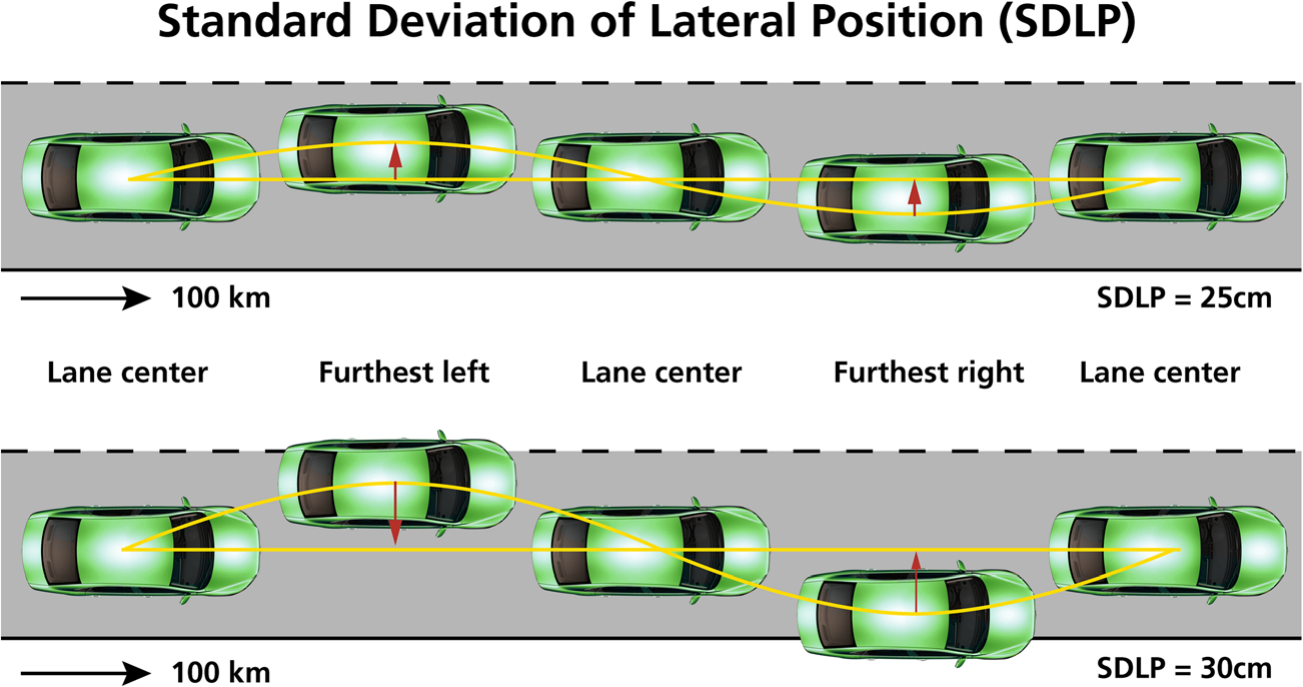
Standard deviation of lateral position (SDLP). SDLP is calculated relative to the mean lateral position over the entire driving test. The bottom figure illustrates that with reduced vehicle control, SDLP values increase. This increased weaving may ultimately lead to excursions out of lane

During screening, subjects were trained once to obtain baseline performance on the driving test. Subjects had to perform a standardized on-the-road driving test (Verster and Roth 2011). They had to operate a specially instrumented vehicle on a public highway during normal traffic on a 100-km track between the cities of Utrecht and Arnhem (the Netherlands). They were instructed to drive with a steady lateral position (as straight as possible) and a constant speed of 95 km/h on the right (slower) traffic lane. They were allowed to overtake a slower driving vehicle in the same traffic lane. A licensed driving instructor who had access to dual controls sat on the passenger’s seat to guard the safety of the participant during the driving test. A computer in the backseat of the car continuously recorded the speed and position of the car within the traffic lane. The standard deviation of the lateral position (SDLP, cm), i.e., the weaving of the car, is the primary outcome measure (Fig. 1). SDLP was calculated after off-line editing of the data that was disturbed by events (e.g., overtaking a slower car, traffic jams). As illustrated by Fig. 1, when vehicle control is reduced (e.g., when sleepy or after using sedative drugs), SDLP values increase (i.e., more weaving of the car). SDLP has been proven to be a robust measure within participants, capable to demonstrate dose-dependent driving impairment (Verster and Mets 2009; Verster and Roth 2011). As a cutoff value for clinically impaired driving impairment, usually one refers to the SDLP increase relative to placebo of +2.4 cm (Louwerens et al. 1987), which was observed when driving after the consumption of alcohol to reach a blood alcohol concentration (BAC) of 0.05%, i.e., the legal limit for driving in many countries.

The secondary parameter was the standard deviation of speed (SDS, km/h). Control variables were mean lateral position (MLP, +/− cm) and mean speed (MS, km/h). Driving tests were stopped before completion if the driving instructor or the participant felt it was unsafe to continue.

### Subjective assessments

The Groningen Sleep Quality Scale (GSQS) is a participant-reported assessment used to assess the quality of the previous night sleep (Mulder-Hajonides van der Meulen WREH 1980). It consists of 15 items, and scores range from 0 to 14.6. In general, if sleep was unrestricted and undisturbed, participants score 0 to 2 points. A higher score (6 to 7) indicate disturbed sleep. The GSQS was completed before treatment administration. The test day was postponed when GSQS scores were higher than 6.

The Karolinska Sleepiness Scale (KSS) is a subject-reported assessment used to rate sleepiness on a scale of 1 to 9, ranging from “extremely alert” (1) to “very sleepy, great effort to keep awake, fighting sleep” (Åkerstedt and Gillberg 1990). The KSS was completed directly before the driving test.

After the driving test, subjects indicated the perceived quality of their driving performance on a scale ranging from “I drove exceptionally poorly” to “I drove exceptionally well” around a midpoint of “I drove normally” (Verster and Roth 2011). The level of effort they had to invest to complete the driving test was indicated on a 15-cm scale, with multiple markings ranging from “absolutely no effort” to “extreme effort” (Zijlstra and van Doorn 1985).

### Sample size

The sample size and power estimation were based upon the SDLP, the primary end point of the study. A non-inferiority margin of 2.4 cm in SDLP (associated with blood alcohol content of 0.05%) that was considered clinically relevant was used for the power calculation. Assuming that the true difference in SDLP between esketamine and placebo was 0.63 cm, with a two-sided significance level of 0.05 (one-sided 0.025) and a SD of a difference in paired case of 2.97 cm (i.e., within-subject SD in a crossover of 2.1 cm), a sample size of 24 subjects achieved 80% power to detect non-inferiority between treatments (one-sided paired *t* test).

### Statistical analysis

The statistical analysis was performed using Statistical Analysis Software (SAS), version 9.2.

Statistical analysis was conducted using an analyses of variance (ANOVA) model with treatment, sequence, period, and gender as fixed effects, and subjects within sequence as a random effect. Pairwise comparisons between the treatments and placebo were conducted. A similar ANOVA model was used for analyses of secondary parameters (SDS, MLP, and MS).

In addition, for SDLP only, a non-inferiority test was conducted. Non-inferiority between each active treatment and placebo was concluded if the upper limit of the two-sided 95% confidence interval of the mean difference between treatment and placebo was <2.4 cm.

## Results

A total of 26 subjects participated in the study. Of these, two subjects did not complete the study. One subject was withdrawn due to an adverse effect (atrial fibrillation, unrelated to the study drug) and the other subject discontinued study participation due to withdrawal of consent. None of the subjects had a total GSQS score greater than 6 on any test day. Demographics of the 24 subjects that were included in the statistical analyses are summarized in Table 1.

**Table 1:** Demographic characteristics of the participants

	Men (*N* = 12)	Women (*N* = 12)	Overall (*N* = 24)
Age (years)	26.3 (8.25)	28.0 (9.01)	27.1 (8.49)
Weight (kg)	80.3 (9.75)	66.2 (9.70)*	73.3 (11.95)
Height (cm)	183.8 (7.73)	170.5 (6.41)*	177.2 (9.72)
BMI (kg/m^2^)	23.7 (2.15)	22.7 (2.04)	23.2 (2.12)

Mean (SD) are shown

*BMI* body mass index, *SD* standard deviation

Significant differences (*p* < 0.05 based on a two-sample *t* test) between men and women

### Driving performance

Results of the driving test are summarized in Table 2. Mean (SE) SDLPs were 17.10 cm (0.92), 19.38 cm (0.91), and 17.25 cm (0.92) after esketamine, mirtazapine, and placebo, respectively. The upper limit of the two-sided 95% CI of the mean difference between esketamine and placebo was +0.86 cm. As this is below the prespecified non-inferiority margin of +2.4 cm, esketamine was non-inferior to placebo. In contrast, the upper limit of the two-sided 95% CI of the mean difference between mirtazapine and placebo (+3.15 cm) was greater than the non-inferiority margin of +2.4 cm.

**Table 2:** Driving test results

	Esketamine (84 mg) Mean (SE)	Mirtazapine (30 mg) Mean (SE)	Placebo Mean (SE)
SDLP (cm)	17.10 (0.92)	19.38 (0.91)*	17.25 (0.92)
SDS (km/h)	2.40 (0.16)	2.49 (0.16)	2.28 (0.16)
MLP (cm)	10.85 (2.57)	8.33 (2.53)	8.73 (2.55)
MS (km/h)	97.96 (0.35)	97.72 (0.34)	97.79 (0.34)

*SDLP* standard deviation of lateral position, *SDS* standard deviation of speed, *MLP* mean lateral position, *MS* mean speed, *SE* standard error

*Significant differences from placebo (*p* < 0.05)

**Fig. 2:**
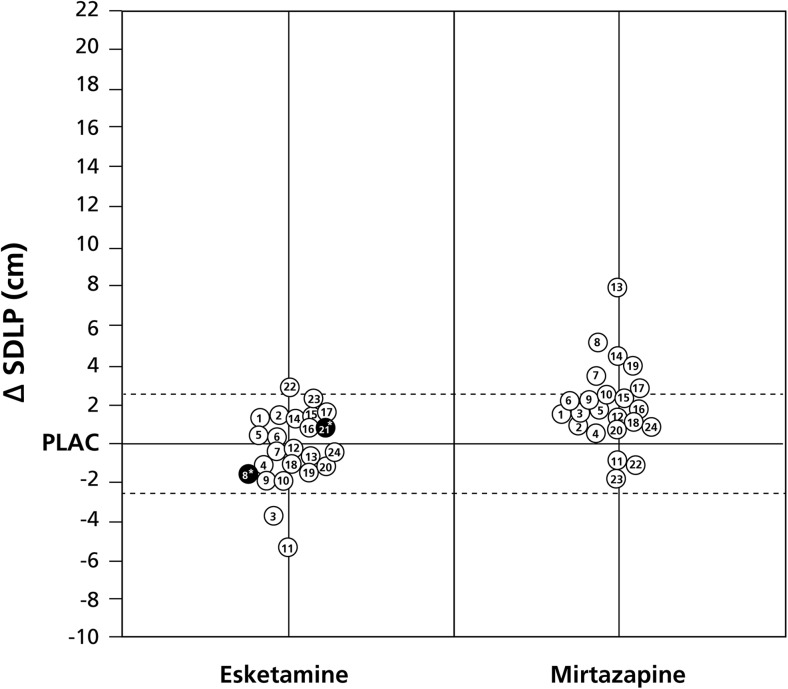
Individual driving performance. Difference scores (∆SDLP, treatment − placebo) are depicted. The *dotted lines* represent the cutoff values for non-inferiority (+2.4 cm) and superiority (−2.4 cm). Same numbers are same subjects. Subjects no. 8 and 21 had driving tests that were stopped before completion (depicted in *black*). Note that subject no. 21 did not perform the driving test in the mirtazapine condition. Abbreviations: *SDLP* standard deviation of lateral position, *PLAC* placebo

No significant differences from placebo were found for standard deviation of speed, as well as the control variables mean speed and mean lateral position. Individual driving performances, i.e., ∆SDLP (treatment − placebo), are summarized in Fig. 2.

### Subjective driving assessment

Compared to placebo (12.10 cm), perceived driving quality was significantly lower after mirtazapine (8.8 cm, *p* = 0.0029), perceived driving quality scores were also lower after esketamine (10.11 cm), but this difference was not statistically significant (*p* = 0.0676).

Compared to placebo (4.58 cm), perceived effort to perform the driving test was significantly higher (more effort) after both esketamine (6.47 cm, *p* = 0.0169) and mirtazapine (6.29 cm, *p* = 0.0278).

### Stopped driving tests

Two subjects did not complete the driving tests after administration of esketamine, due to adverse events. One of these subjects (no. 21 in Fig. 2) also declined the driving test with mirtazapine due to ongoing nausea and dizziness. This subject reported paresthesia and headache during the driving test after esketamine. Although the driving of the subject was assessed as safe by the driving instructor, the subject stopped the driving test after 46 km. The second subject who discontinued the driving test (no. 8 in Fig. 2) experienced blinding by the traffic lights which could be associated with esketamine or underlying migraine. For this subject, driving was assessed as impaired by the driving instructor, with a potential safety impact, and the test was stopped after 35 km. This subject had also reported the adverse event of migraine in the prior study period (after administration of placebo). Importantly, both subjects timely verbalized their wish to stop the test and reported their adverse events, thus minimizing any risk of accident. As is evident from Fig. 2, both subjects’ actual driving performance before stopping the test, as expressed in ∆SDLP, was below the upper limit of the prespecified non-inferiority margin of +2.4 cm.

### Blood plasma concentrations

All subjects exhibited measurable concentrations of esketamine, noresketamine, and mirtazapine prior to the driving test. Plasma concentrations of esketamine, noresketamine, and mirtazapine were 15.4 ng/mL (4.81), 80.9 ng/mL (32.7), and 28.0 ng/mL (8.45), respectively. Non-parametric Spearman’s *r* correlations between ∆SDLP (treatment − placebo) and plasma drug concentration were not significant for esketamine (*r* = −0.243, *p* = 0.253), noresketamine (*r* = −0.294, *p* = 0.163), and mirtazapine (*r* = 0.258, *p* = 0.235).

### Adverse effects

The incidence of treatment-emergent adverse events was higher after esketamine (100.0%) compared to mirtazapine (88.5%) and placebo (37.5%). After esketamine, the most frequently reported adverse events were dizziness (66.7%), dissociation (50.0%), nausea (45.8%), paraesthesia (45.8%), headache (45.8%), dysgeusia (29.2%), feeling drunk (29.2%), and fatigue, vomiting and somnolence, euphoric mood, and blurred vision (each reported by 20.8% of participants). Most of the adverse events observed after esketamine were mild in severity, transient, and resolved within 2–3 h after dosing. After mirtazapine, the most frequently (≥20%) reported adverse events were somnolence (65.4%) and fatigue (26.9%), typically lasting several hours to several days. After placebo, the most commonly reported adverse event was headache (12.5%).

## Discussion

On-road driving performance, as measured by SDLP, was not significantly impaired 8 h after intranasal administration of esketamine (84 mg). The upper limit of the two-sided 95% CI of the mean difference between esketamine and placebo was +0.86 cm, which is above the non-inferiority margin of +2.4 cm. Hence, it can be concluded that esketamine was not inferior to driving after placebo. In contrast, driving was significantly impaired 8 h after oral administration of mirtazapine (30 mg). The mean difference between mirtazapine and placebo was +3.15 cm, well above the upper limit of the two-sided 95% CI. As mirtazapine served as positive control, the latter was anticipated and the results confirm assay sensitivity.

While this is the first study investigating driving performance after esketamine, several studies have examined mirtazapine’s effect on on-road driving. Verster et al. (2015) reviewed three on-road and four driving simulator studies, which revealed dose-dependent driving impairment the morning following bedtime administration of mirtazapine (15 or 30 mg). The observed magnitude of driving impairment relative to placebo was comparable to that seen when driving with a BAC of 0.05%. Hence, mirtazapine is a suitable comparator (positive control) in driving tests examining antidepressant drugs. Of note, whereas in the previous studies mirtazapine was administered at bedtime and after a full night of sleep driving tests were performed in the morning, in the current study the drug was administered in the morning and driving tests were in the early evening, with no opportunity to sleep during the day.

Two driving tests after treatment with esketamine were stopped before completion, and another driving test was not started after treatment with mirtazapine. It is sometimes suggested that when driving tests are stopped before completion this is evidence that a drug is unsafe. However, the number of stopped tests is actually a poor predictor of a drug’s effects on driving performance. In fact, a review of 50 driving studies revealed that in 14 of these clinical trials driving tests are also stopped after treatment with placebo (Verster and Roth 2012).

It is important to examine in detail the causes of premature stopping the respective driving tests. Stopping a driving test is a subjective decision that depends on the awareness of performance impairment and risk perception by either the driver or the driving instructor (Verster and Roth 2012). Sometimes, a clear association with drug effects is evident, for example, if drivers are very sleepy due to administering drugs with sedative properties. However, drivers themselves can have many other reasons for stopping the driving tests. These may not be related to treatment-emergent adverse effects or to actual performance impairment. In other words, tests can be stopped if no significant increase in SDLP is observed and no takeover maneuvers were needed by the driving instructor (Verster and Roth 2012). Therefore, it may be concluded that stopped driving tests are a poor indicator to what extent it is safe to drive with a certain drug. Also in the current study, actual driving performance in the two stopped driving tests was within the non-inferiority boundaries (Fig. 2). One of the subjects who stopped her driving test after esketamine seems more sensitive to drug effects than most other participants, as she also refused to start her driving test after mirtazapine. Taken together, although two driving tests were stopped before completion, this should not be viewed as supportive evidence that driving after esketamine is unsafe.

Blood plasma concentrations of esketamine, noresketamine, and mirtazapine did not correlate significantly with performance on the driving test (i.e., treatment − placebo differences). Driving is an example of complex behavior, and at baseline large differences can be seen between individual drivers, as expressed in SDLP values ranging from around 10 to 30 cm (Verster and Roth 2011). However, the absolute difference in SDLP (treatment − placebo) is fairly constant among drivers with different baseline SDLPs (Verster and Roth 2011), despite sometimes large differences in blood drug concentrations. A similar poor relationship between blood drug concentration and individual ∆SDLPs has been reported for benzodiazepine drugs (Verster and Roth 2013). As expected, esketamine treatment was associated with adverse events related to nervous system and psychiatric disorders in nearly all patients. However, these adverse events were transient and resolved in most patients within 2–3 h after dosing including dissociative symptoms which was observed in 13 (50%) patients.

Previous reports suggest that the most common adverse events associated with esketamine treatment are drowsiness, dizziness, drunkenness, sensation of floating, distorted body experience, and distorted vision/hearing (Persson et al. 2002; Segmiller et al. 2013; Vollenweider et al. 1997). Consistent with this, the most frequently reported adverse events in the present study were dizziness, dissociation, nausea, paraesthesia, headache, dysgeusia, feeling drunk, fatigue, vomiting and somnolence, euphoric mood, and blurred vision. There were no new or unexpected safety concerns noted with the administration of intranasal esketamine during the study.

The current study has several potential limitations that should be addressed. First, in this study driving tests were performed in the evening, after being awake during the day, 8 h after treatment administration. This may have had a negative impact on the participants’ alertness levels and sleepiness. Also contributing to increased sleepiness was the fact that tests were conducted after sundown. However, as this was a cross-over trial, possible effects of evening driving would have been experienced equally on each test day and patients taking these medications could have to drive in the evening or after dark in real-world conditions. Second, as only a single dose of each treatment was administered, it is unknown if driving performance improves after repeated treatment administration. For many drugs, tolerance is seen after repeated drug use in terms that users experience less side effects and driving performance slowly returns to baseline (placebo) performance levels. This is for example seen after repeated administration of mirtazapine (Verster et al. 2015). Future studies should therefore examine the effects on driving ability of sub-chronic use of esketamine. In the current study, the time between treatment administration and driving was 8 h. If indeed after sub-chronic use tolerance develops to the effects of esketamine, the time between treatment administration and driving may be reduced, without compromising safety. However, this needs to be confirmed in future studies.

A limitation may be regarded that the current study was conducted in healthy volunteers as opposed to patients with major depression. This was however not a limitation but done on purpose. If one is interested in the pharmacological effects of a drug on driving, healthy volunteers should be tested. That way, assessments are not affected by health- or disease-related factors. For example, in case assessments were made in depressed patients instead of healthy participants, drug-disease interactions could obscure the study outcome in that adverse drug effects may be reversed by possible treatment effects (i.e., a reduction in depression scores). It is then difficult to interpret the study outcome. If a drug has been investigated in healthy volunteers, a next step may be to further examine its effects on driving in the intended patient population. Also for esketamine, it would be interesting to examine its effects in depressed patients, both after single-dose administration and after sub-chronic use. Finally, as esketamine was administered in the morning and driving tests were performed in the evening, it may be interesting if the absence of effects on driving performance is also seen when esketamine is administered in the evening and the driving tests are performed next morning. Future research should address these topics.

In summary, administration of intranasal esketamine did not significantly impair driving performance 8 h thereafter. Future research should confirm these findings in patients with major depression, and investigate the effects of chronic esketamine use.
